# Atomistic and electronic insights into Ca^2+^ and Li^+^ intercalation in TiS_2_: a first-principles approach supported by electrochemical validation

**DOI:** 10.1038/s41598-026-42087-w

**Published:** 2026-03-23

**Authors:** Seunga Yang, Sangyup Lee, Paul Maldonado Nogales, Yangsoo Kim, Soon-Ki Jeong

**Affiliations:** 1 Department of Future Convergence Technology, Graduate School, Soonchunhyang University, Soonchunhyang- ro 22-gil, Sinchang-myeon, Asan-si, 31538 Chungcheongnam-do Republic of Korea; 2 Jeonju Center, Korea Basic Science Institute, Jeonju-si, 54907 Jeollabuk-do Republic of Korea; 3 Department of Energy Engineering, Soonchunhyang University, Soonchunhyang-ro 22-gil, Sinchang-myeon, Asan-si, 31538 Chungcheongnam-do Republic of Korea; 4 Advanced Energy Research Center, Soonchunhyang University, Soonchunhyang-ro 22-gil, Sinchang-myeon, Asan-si, 31538 Chungcheongnam-do Republic of Korea

**Keywords:** Ca-ion batteries, Structural stability, First-principles calculations, TiS_2_ (titanium disulfide), Chemistry, Energy science and technology, Materials science

## Abstract

Calcium-ion batteries are emerging as a sustainable and high-energy alternative to lithium systems, yet the atomic-scale origin of their ion–host interactions remains unclear. We clarified the coupling between ion mobility and electronic structure in titanium disulfide (TiS_2_) by combining multiscale density functional theory with experimental analysis. Periodic VASP simulations and localized DV-Xα analyses revealed that Ca^2+^ intercalation induces greater lattice expansion than Li^+^, lowers diffusion barriers, and enriches the density of states near the Fermi level, enhancing both ionic and electronic transport. Despite weaker Ca–S interactions, strong Ti–S covalency stabilizes the framework, yielding a theoretical open-circuit voltage of 1.383 V, which is lower than that of LiTiS_2_ (1.948 V). Orbital overlap and charge-transfer analyses show that this lower voltage reflects a balance between multi-electron charge storage (z = 2 for Ca^2+^) and moderated electronic restructuring, rather than a simple reduction in electrochemical performance. Electrochemical measurements confirm these results: Ca-intercalated TiS_2_ delivers a first-cycle capacity of 201 mAh·g^− 1^, superior diffusion coefficients, and 96.3% rate retention with stable cycling. This work provides the first atomistic evidence that Ca^2+^ insertion facilitates ion transport while imparting structural resilience, offering a design framework for next-generation multivalent-ion batteries.

**Supplementary Information:**

The online version contains supplementary material available at 10.1038/s41598-026-42087-w.

## Introduction

Lithium-ion batteries (LIBs) have long served as the cornerstone of modern energy-storage technology for portable electronics and electric vehicles owing to their high energy density, long cycle life, and excellent reversibility^1–3^. However, the growing demand for batteries with higher energy density, coupled with the limited availability of lithium resources and ongoing safety concerns, has intensified the search for next-generation systems^4–7^. Among the alternatives, multivalent-ion batteries (MIBs) that utilize charge carriers such as Mg^2+^, Ca^2+^, Zn^2+^, and Al^3+^ are receiving considerable attention. Because multivalent ions can transfer multiple electrons per ion, they can theoretically store more charge than conventional monovalent-ion systems^8,9^.

Among MIBs, calcium-ion batteries (CIBs) are particularly promising. The standard reduction potential of Ca^2+^ (− 2.87 V vs. SHE) is close to that of Li^+^ (− 3.05 V), yet Ca^2+^ is a divalent charge carrier^10–14^. This combination of high operating voltage and divalent charge transport implies that CIBs can achieve energy densities comparable to—or even surpassing— those of LIBs^15,16^. Furthermore, calcium is naturally abundant, low cost, and environmentally benign, which enhances its potential for large-scale and sustainable energy-storage applications. Despite these advantages, the fundamental understanding of how Ca^2+^ interacts with host materials—particularly in terms of bonding, diffusion, and electronic structure—remains incomplete, hindering further progress in this field.

In pursuit of suitable electrode materials for CIBs, layered transition-metal dichalcogenides such as TiS_2_, MoS_2_, and WS_2_ have emerged as attractive candidates owing to their two-dimensional frameworks that can accommodate large cations^17–19^. Among them, TiS_2_ is particularly notable for its narrow bandgap and wide interlayer spacing (0.57 nm), which is considerably larger than that of graphite (0.357 nm)^20–27^. These structural characteristics facilitate ion intercalation and diffusion, reduce energy barriers for insertion, and maintain lattice stability during cycling. As such, TiS_2_ provides a well-defined model platform for exploring ion–host interactions in multivalent-ion systems.

In parallel, TiS_2_ has also been actively investigated as a cathode material for lithium-ion batteries, where its layered structure enables rapid Li^+^ transport through van der Waals gaps and reversible intercalation without severe lattice collapse. Recent studies have shown that Li^+^ insertion in TiS_2_ has been reported to exhibit different mechanistic features, including solid-solution-type behavior as well as phase-transition-related (two-phase) characteristics, depending on electrochemical conditions, accompanied by reversible Ti redox reactions and relatively fast reaction kinetics^28–30^. Spectroscopic and theoretical analyses further revealed that strong hybridization between Ti 3d and S 3p orbitals and the intrinsically high electronic conductivity of TiS_2_ play key roles in governing Li^+^ intercalation energetics^31,32^. At the same time, intrinsic limitations such as modest operating voltage and gradual structural or interfacial degradation during prolonged cycling have been reported in lithium-based TiS_2_ systems^33,34^. However, these accumulated Li-ion studies establish TiS_2_ as a well-characterized monovalent-ion host, providing a solid reference framework for extending investigations toward multivalent-ion intercalation.

Nevertheless, studies on Ca-ion intercalation in TiS_2_ remain scarce, with only a few published to date. Tchitchekova et al. first demonstrated Ca^2+^ intercalation into TiS_2_ at elevated temperatures using a Ca(BF_4_)_2_-based electrolyte^35^. Subsequently, our group successfully inserted Ca ions at room temperature using a Ca(CF_3_SO_3_)_2_-based electrolyte, demonstrating its practicality^36^. To further assess the system’s applicability under broader electrochemical environments, the investigation was extended to aqueous high-concentration electrolytes^37^. While these studies verified the feasibility of Ca-ion storage, they offered limited insight into atomic-level processes such as ion migration, charge transfer, and structural evolution, which are essential to understanding and improving long-term performance. In this context, Li^+^ intercalation in TiS_2_ is intentionally employed in this work as a well-established monovalent reference system with extensively studied thermodynamic and kinetic characteristics. By directly comparing Li^+^ and Ca^2+^ intercalation within an identical TiS_2_ host lattice, this study aims to decouple the effects of ionic valence and charge compensation on adsorption energetics, diffusion barriers, and electronic-structure evolution. Such a comparative framework enables Ca^2+^-specific behaviors to be interpreted against a clear physical baseline, thereby clarifying the unique challenges and opportunities associated with divalent-ion intercalation beyond what can be inferred from Ca-only investigations.

In this work, theoretical and experimental approaches are combined to investigate the structural, electronic, and kinetic behavior of Li^+^ and Ca^2+^ intercalation in TiS_2_. Density functional theory (DFT) and discrete variational Xα (DV-Xα) methods were employed to analyze diffusion barriers, bonding topology, and charge redistribution. The theoretical predictions were validated through galvanostatic cycling and cyclic voltammetry (CV) measurements, establishing direct correlations between atomic-scale interactions and electrochemical performance. This integrated framework not only bridges the gap between existing theoretical and experimental studies but also provides atomic-level design principles for developing durable and high-performance CIBs.

## Methods

### Cluster model setup for intercalation studies

To investigate the intercalation behavior of Li^+^ and Ca^2+^ ions in TiS_2_, a finite cluster model was constructed to approximate the local coordination geometry and electronic environment of bulk TiS_2_. This cluster-based approach enables localized analysis of interfacial bonding, ion diffusion mechanisms, and structural perturbations during intercalation. The model comprises 26 Ti atoms and 48 S atoms, corresponding to a representative segment of the layered TiS_2_ framework. To simulate ion-insertion conditions, 13 guest ions (Li^+^ or Ca^2+^) were introduced into the interlayer spacing, maintaining a stoichiometric ratio consistent with experimental intercalation levels. The charge state of the system was adjusted to reflect the formal oxidation states of the host and guest species, preserving overall charge neutrality. This configuration permits a direct comparison of Li and Ca intercalation under equivalent structural constraints and enables detailed evaluation of site-specific bonding and mobility.

### Structural optimization using VASP

First-principles density functional theory (DFT) calculations were performed using the Vienna Ab Initio Simulation Package 5.4 (VASP) to optimize the atomic structures of LiTiS_2_ and CaTiS_2_. Structural relaxation accounted for volume changes and lattice distortions induced by ion intercalation, providing a reliable foundation for electronic structure analysis. The exchange–correlation energy was treated using the generalized gradient approximation with the Perdew–Burke–Ernzerhof functional, striking a balance between computational efficiency and accuracy. A Monkhorst–Pack k-point mesh of 9 × 9 × 3 was employed with a 500 eV plane-wave cutoff, and convergence criteria were set to an energy difference below 10^− 5^ eV and atomic forces below 0.01 eV·Å^−1^. No explicit long-range dispersion (vdW) correction (e.g., DFT-D3 or vdW-DF) was applied in the present calculations. Although vdW interactions can contribute to interlayer binding in pristine layered materials, the optimized lattice parameters of pristine TiS_2_ obtained under our computational settings agree well with reported experimental values (Sect.  3.1). In ion-intercalated TiS_2_, the interlayer interaction arises from a combination of electrostatic, covalent, and dispersion contributions. Because the present study is intended to compare Li^+^ and Ca^2+^ intercalation under identical settings, the calculated structures and electronic descriptors are interpreted primarily in terms of relative trends rather than quantitative interlayer binding energies. In this study, the electronic-structure analysis is primarily focused on charge-transfer-driven bonding evolution associated with guest-ion insertion, which has been widely adopted as an effective framework for understanding ion-intercalated layered materials in previous first-principles studies^38–40^. The fully relaxed geometries from VASP then served as input for DV-Xα calculations, ensuring consistency between structural and electronic analyses and enabling a comprehensive evaluation of ion-induced structural evolution for CIB performance.

### Electronic state and bonding analysis using DV-Xα

To investigate the electronic structure and bonding characteristics of TiS_2_, the DV-Xα method was employed, a self-consistent molecular orbital (MO) approach based on local density approximation. This technique enables orbital-level analysis of bonding and charge distribution, offering atomic-scale insights into ion–host interactions and conductivity. Orbital overlap between guest ions (Li^+^ or Ca^2+^) and host atoms (S and Ti) was examined to assess bonding strength, hybridization, and implications for ionic mobility. Density of states (DOS), effective charges, and covalent electron populations were additionally calculated to characterize electronic states, charge transfer, and their influence on diffusion barriers. Bandgaps were estimated using the “lvlshm” command to link electronic excitation with charge transport. These results complement the DFT results, providing a comprehensive understanding of both ionic and electronic processes in TiS_2_.

### Band structure and OCV analysis using VASP

The electronic band structure and theoretical open-circuit voltage (OCV) of Li- and Ca-intercalated TiS_2_ systems were calculated using first-principles DFT, as implemented in VASP. A plane-wave cutoff energy of 500 eV was employed, and the energy-convergence criterion for ionic relaxation was set to 0.05 eV·Å^−1^ to ensure numerical stability. Band structure calculations were conducted along high-symmetry paths in the Brillouin zone using the optimized unit cell geometries, enabling analysis of the electronic bandgap and carrier mobility.

All band-structure calculations for pristine, Li-intercalated, and Ca-intercalated TiS_2_ were performed within the same PBE (GGA) + U framework using identical computational parameters, enabling direct comparison of electronic-structure changes induced by different intercalating ions. Within this consistent computational setting, the calculated band structures are used to analyze relative features such as band dispersion, band-edge shifts, and band-gap evolution associated with ion intercalation^41–43^. In this context, the band-gap values are discussed as descriptors of electronic-structure evolution under a unified theoretical framework, providing insight into how Li^+^ and Ca^2+^ intercalation differently modulate the electronic states of TiS_2_.

The OCV was computed using the total energies of the intercalated and pristine structures, based on the following expression^44,45^:

$$OCV=-\frac{\left[\right\{E\left({\mathrm{L}\mathrm{i}}_{n}\mathrm{T}\mathrm{i}{\mathrm{S}}_{2}or{\mathrm{C}\mathrm{a}}_{n}\mathrm{T}\mathrm{i}{\mathrm{S}}_{2}\right)-E\left({\mathrm{L}\mathrm{i}}_{m}\mathrm{T}\mathrm{i}{\mathrm{S}}_{2}or{\mathrm{C}\mathrm{a}}_{m}\mathrm{T}\mathrm{i}{\mathrm{S}}_{2}\right)-\left(n-m\right)E\left({\mathrm{L}\mathrm{i}}_{bulk}\mathrm{o}\mathrm{r}{\mathrm{C}\mathrm{a}}_{bulk}\right)}{(n-m)\times\mathrm{z}}$$

where *E*(Li_*n*_TiS_2_ or Ca_*n*_TiS_2_) and *E*(Li_*m*_TiS_2_ or Ca_*m*_TiS_2_) represent the total energies of the intercalated and less-intercalated states, respectively, and *E*(Li_bulk_ or Ca_bulk_) is the reference energy per Li or Ca atom, obtained by calculating the total energy of the corresponding bulk metallic crystal and normalizing it by the number of metal atoms in the unit cell. Here, z denotes the valence charge of the intercalating ion ($z=1$ for Li^+^ and z = 2 for Ca^2+^), reflecting the number of electrons transferred per inserted ion. As the total energies are expressed in eV per formula unit, the resulting value corresponds directly to the potential in volts; therefore, an explicit Faraday constant is not required. This formulation enables a direct and quantitative comparison of the electrochemical potentials of LiTiS_2_ and CaTiS_2_.

### Adsorption and diffusion energy calculations

The adsorption energy (*E*_ads_​) of Li^+^ and Ca^2+^ ions on TiS_2_ surfaces was calculated using a standard expression widely employed in surface science^46^:

$${E}_{\mathrm{a}\mathrm{d}\mathrm{s}}={E}_{{\mathrm{T}\mathrm{i}\mathrm{S}}_{2}+\mathrm{i}\mathrm{o}\mathrm{n}}-{E}_{{\mathrm{T}\mathrm{i}\mathrm{S}}_{2}}-{E}_{\mathrm{g}\mathrm{u}\mathrm{e}\mathrm{s}\mathrm{t}\mathrm{i}\mathrm{o}\mathrm{n}\left(\mathrm{b}\mathrm{u}\mathrm{l}\mathrm{k}\right)}$$

where ${E}_{{\mathrm{T}\mathrm{i}\mathrm{S}}_{2}+\mathrm{i}\mathrm{o}\mathrm{n}}$ is the total energy of the TiS_2_ structure with an adsorbed Li^+^ or Ca^2+^ ion, ${E}_{{\mathrm{T}\mathrm{i}\mathrm{S}}_{2}}$is the total energy of the pristine TiS_2_ surface, and *E*_guest ion (bulk)_ refers to the energy of Li or Ca in the bulk metallic state; the more negative the *E*_ads_ value, the more thermodynamically stable the ion host interface.

The diffusion energy barrier (*E*_diff_) was evaluated by determining the minimum energy path between adjacent adsorption sites using the nudged elastic band (NEB) method^47^:

$${E}_{\mathrm{d}\mathrm{i}\mathrm{f}\mathrm{f}}={E}_{\mathrm{T}\mathrm{S}}-{E}_{\mathrm{i}\mathrm{n}\mathrm{i}\mathrm{t}\mathrm{i}\mathrm{a}\mathrm{l}}$$

where *E*_initial_ represents the total energy of the initial adsorption state, and *E*_TS_ denotes the energy of the transition state corresponding to the highest energy point along the diffusion pathway. The transition state is defined as the first-order saddle point on the potential energy surface, at which the atomic configuration experiences a maximum in energy along the reaction coordinate but remains locally stable in all perpendicular directions. This state therefore represents the kinetic bottleneck that governs ion migration within the host lattice^48^. Accordingly, *E*_diff_ corresponds to the activation energy required for ion diffusion, and its magnitude directly determines the rate of ion transport. A lower *E*_diff_ value indicates reduced kinetic resistance, leading to more facile ion migration and enhanced rate capability in electrochemical systems^49–52^.

### Experimental methods

#### Fabrication of TiS_2_ composite electrodes

TiS_2_ powder (Sigma-Aldrich, St. Louis, MO, USA), Super P conductive carbon (TIMCAL, Bodio, Switzerland), and poly(vinylidene fluoride) (Mw ≈ 534,000; Alfa Aesar, Haverhill, MA, USA) were mixed in a weight ratio of 7:2:1. The mixture was dispersed in *N*-methyl-2-pyrrolidone (Junsei Chemical, Tokyo, Japan) to form a homogeneous slurry. The TiS_2_ powder used in this study was obtained from a commercial supplier (Sigma-Aldrich) and was used as received. The layered TiS_2_ phase has been extensively characterized in prior studies using X-ray diffraction and Raman spectroscopy, which report characteristic diffraction peaks and vibrational modes under ambient conditions^29,53,54^. Accordingly, we cite these prior structural reports for phase identification, while the present work focuses on electrochemical evaluation under moisture-controlled, nonaqueous conditions. This slurry was cast onto 18 μm thick copper foil (UACJ Corporation, Tokyo, Japan) using a doctor blade and dried in a vacuum oven (OV-11, Jeio Tech, Daejeon, Republic of Korea) at 80 °C for 12 h. The dried electrodes were punched into 15.95 mm discs and transferred into an Ar-filled glove box (SK-G1200, Three-Shine, Daejeon, Republic of Korea) with H_2_O and O_2_ levels maintained below 0.5 ppm.

#### Electrochemical evaluation

CR2032-type coin cells (Welcos, Daejeon, Republic of Korea) were assembled in an Ar-filled glove box (SK-G1200, Three-Shine). The TiS_2_ composite electrode served as the working electrode, while lithium metal foil (Honjo Metal, Osaka, Japan) was used as both the counter and reference electrodes. The use of Li metal as a pseudo-reference electrode is considered reasonable in Ca^2+^-based systems, as Ca metal is potentially unstable and exhibits high interfacial impedance^55^. A microporous polypropylene membrane (Celgard 2400, Celgard, Charlotte, NC, USA) was used as the separator. The electrolyte consisted of 0.1 mol dm^− 3^ Ca(TFSI)_2_ (99% purity, TCI, Tokyo, Japan) dissolved in a 1:1 (v/v) mixture of ethylene carbonate and dimethyl carbonate (battery grade, ENCHEM, Cheonan, Republic of Korea). Galvanostatic charge–discharge tests were performed using a battery cycler (WBCS 3000, WonATech, Seoul, Republic of Korea) within a voltage range of 1.5–3.0 V at room temperature. C-rate tests were performed sequentially at 0.1 C, 1 C, 3 C, 5 C, and 10 C, followed by a return to 0.1 C to evaluate rate capability. CV measurements were conducted at varying scan rates of 0.2, 0.5, 0.7, and 1.0 mV·s^− 1^ to analyze the redox behavior and diffusion-controlled kinetics of Li^+^ and Ca^2+^ intercalation in the TiS_2_ host.

## Results

### Structural properties of LiTiS_2_ and CaTiS_2_

**Fig. 1:**
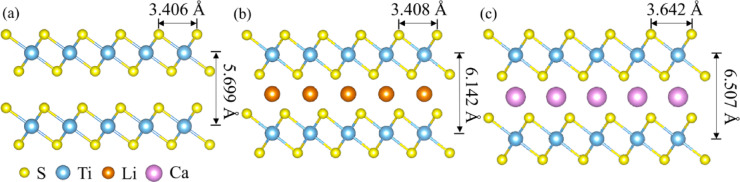
Structural schematics of (**a**) TiS_2_, (**b**) LiTiS_2_, and (**c**) CaTiS_2_ in the (100) plane, highlighting the lattice parameters *a* and *c* with inserted guest ions.

The lattice parameters of pristine TiS_2_ and its Li- and Ca-intercalated derivatives were obtained from VASP-based structural optimization and are summarized in Fig. 1. The initial lattice parameters were based on experimental data reported by Whittingham et al.^56^, ensuring accurate structural modeling. In TiS_2_ (Fig. 1a), the lattice parameter *a*, representing the in-plane S–S distance, is 3.406 Å, while the interlayer spacing, *c*, is 5.699 Å. Both agree well with the experimental values. For LiTiS_2_ (Fig. 1b), *a* increases slightly to 3.408 Å and *c* to 6.142 Å, indicating minor expansion owing to Li-ion intercalation. By contrast, CaTiS_2_ (Fig. 1c) exhibits more substantial increases, with *a* and *c* reaching 3.642 and 6.507 Å, respectively. All structures were modeled with P-3m1 (164) symmetry, ensuring consistency.

**Fig. 2:**
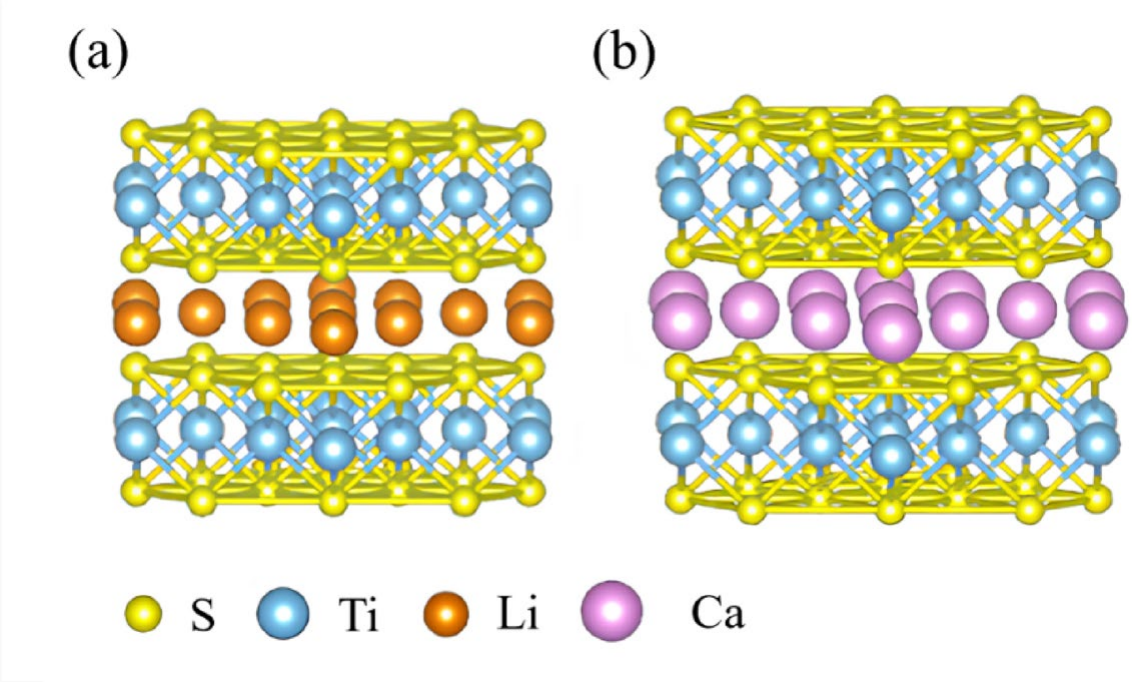
Cluster structures of (**a**) LiTiS_2_ and (**b**) CaTiS_2_ with guest ions situated between two layers in the (100) plane.

Atomic-scale interactions in guest-ion-intercalated TiS_2_ were examined using cluster models constructed for DV-Xα calculations. Based on these optimized structures, local bonding interactions and electron redistribution were further investigated. VASP provided structural optimization and total energy calculations, while DV-Xα offered complementary insights into localized bonding and hybridization. The clusters contained 1 mol of Li or Ca ions, with Li/Ca and Ti atoms aligned along the z-axis and S layers symmetrically positioned on either side. This setup simulated the layered bulk structure for comparative analysis (Fig. 2). Owing to the larger ionic radius of Ca^2+^, the Ca-intercalated cluster exhibits a more pronounced interlayer expansion than its Li-intercalated counterpart.

Following the bonding analysis, various cluster structures were considered, including XTi_2_S_12_, X_7_Ti_14_S_24_, X_7_Ti_14_S_48_, X_13_Ti_14_S_48_, and X_13_Ti_26_S_48_ (X = Li or Ca) (Fig. S1). Among these, the X_13_Ti_26_S_48_ structure was selected because it retains the 1:2 Ti: S ratio after ion insertion, maintaining charge balance for accurate modeling (Table S1). To evaluate electron distribution, the Madelung potential was applied to assign point charges: +1 for Li, + 3 for Ti, and − 2 for S in LiTiS_2_; and + 2 for Ca, + 2 for Ti, and − 2 for S in CaTiS_2_. These values were introduced solely to construct the Madelung embedding potential and to define a consistent electrostatic reference for the DV‑Xα population analysis. Accordingly, they should not be interpreted as representing macroscopic electrode charging or net charge accumulation during battery operation. To avoid confusion, explicit net‑charge annotations for the finite clusters are omitted, and the DV‑Xα results are discussed in terms of relative electron redistribution and bonding evolution within the Ti–S framework upon ion intercalation. This approach reflects the ionic nature of the layered materials and provides a physically consistent description of the local electrostatic environment in the embedded‑cluster analysis.

### Energy-storage properties and overlap of partial DOS

Following the structural analysis, the electronic properties were investigated to gain a deeper understanding of the influence of Li and Ca ions on the electronic behavior of TiS_2_. The total/partial DOS and orbital-overlap characteristics were analyzed to provide a quantitative description of the intercalation-induced electronic-state evolution^57^.

**Fig. 3:**
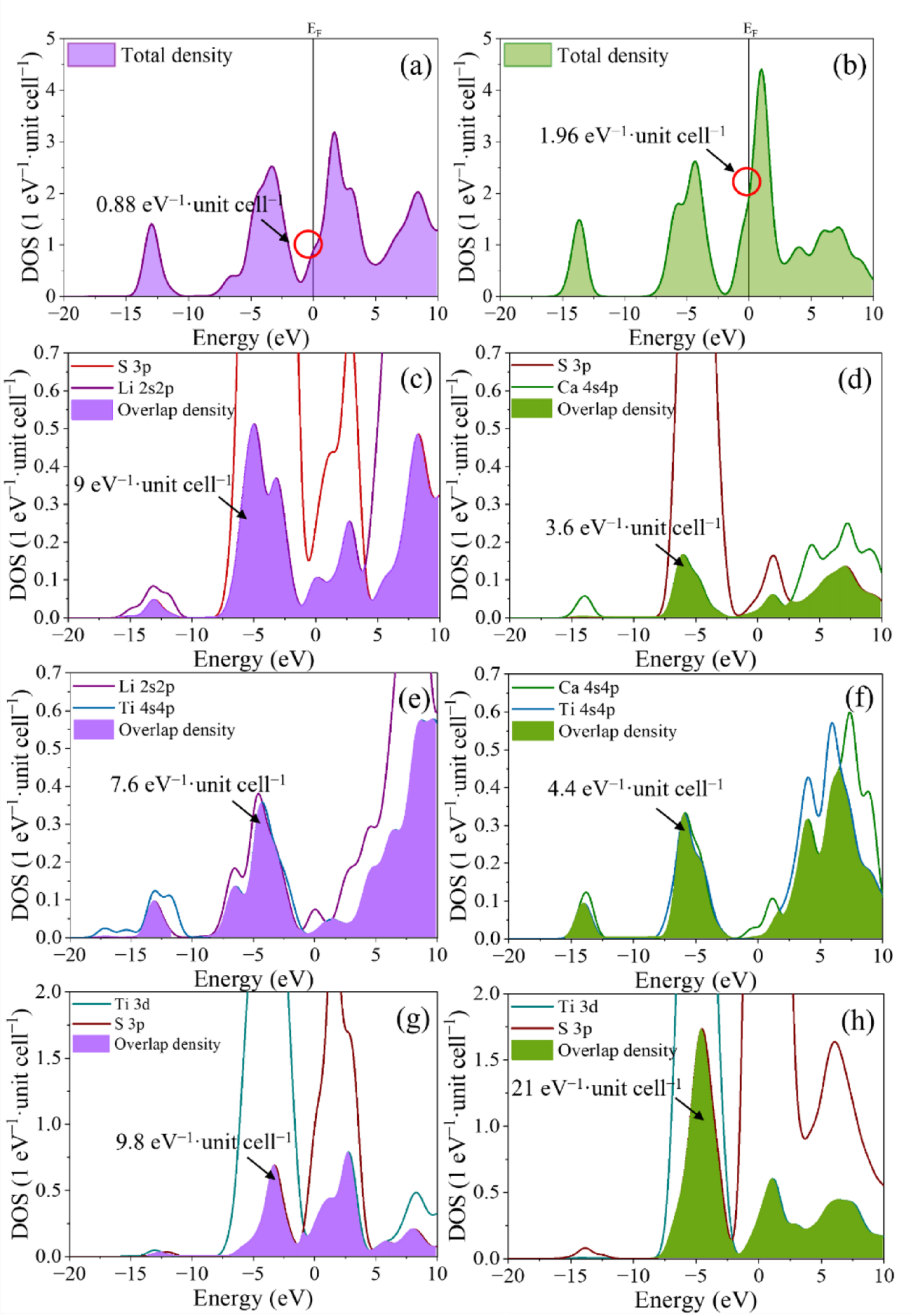
Comparison of DOS: (**a**) total DOS for the Li-intercalated structure Li_13_Ti_26_S_48_; (**b**) total DOS for the Ca-intercalated structure Ca_13_Ti_26_S_48_; partial DOS of the guest-ion sp and S 3p orbitals in (**c**) Li_13_Ti_26_S_48_ and (**d**) Ca_13_Ti_26_S_48_; partial DOS of the guest-ion sp and Ti 4s4p orbitals in (**e**) Li_13_Ti_26_S_48_ and (**f**) Ca_13_Ti_26_S_48_; and partial DOS of the S 3p and Ti 3d orbitals in (**g**) Li_13_Ti_26_S_48_ and (**h**) Ca_13_Ti_26_S_48_.

The DOS analysis was conducted by decomposing contributions from different orbital types, and bonding interactions were evaluated through orbital overlap density calculations, which measure the degree of electron sharing between atomic orbitals. A comparison of the total DOS reveals distinct electronic responses between LiTiS_2_ and CaTiS_2_, particularly near the Fermi level (Fig. 3). The electron density at the Fermi level (E_F_) for the LiTiS_2_ cluster was measured to be only 0.88 eV^− 1^·unit cell^− 1^, in contrast to 1.96 eV^− 1^·unit cell^− 1^ for the CaTiS_2_ cluster. This marked increase in DOS at E_F_ indicates that Ca intercalation modifies the electronic band structure and increases the density of states near E_F_ (Fig. S2)^58,59^.

To further investigate the bonding interactions between the guest ions and the host material, the orbital overlap densities were calculated from the partial DOS. Guest-ion–S interactions were assessed using orbital-overlap densities, which indicate the extent of electron sharing between the guest ion and sulfur. The overlap densities of 9 and 3.6 eV^− 1^·unit cell^− 1^ for LiTiS_2_ and CaTiS_2_, respectively, indicate weak bonding between Ca^2+^ and S atoms (Fig. S3). The corresponding guest-ion–Ti overlap further differentiates the two intercalation chemistries, with Ca–Ti exhibiting a lower overlap than Li–Ti, where the overlap density for Ca–Ti interactions is 4.4 eV^− 1^·unit cell^− 1^, lower than that of Li–Ti (7.6 eV^− 1^·unit cell^− 1^). The orbital overlap density for Ti–S bonding is substantially higher in CaTiS_2_ (21 eV^− 1^·unit cell^− 1^) than in LiTiS_2_ (9.8 eV^− 1^·unit cell^− 1^). This lower orbital overlap reflects weaker ion–host binding strength (Fig. S4), which reduces anchoring effects but may also compromise local structural rigidity, emphasizing the trade-off between ion mobility and framework stability^60^.

### Charge-transfer interactions in TiS_2_ interlayers

The influence of Li and Ca ions on charge-transfer interactions in TiS_2_ interlayers was examined. To quantify the charge redistribution induced by intercalated ions during ion insertion (intercalation) within the interlayer galleries, the effective electron transfer from both guest ions and transition metals as well as sulfur atoms within the TiS_2_ lattice was calculated. Here, the net charge derived from DV-Xα calculations refers to the effective charge associated with electron gain or loss due to partial and delocalized electron redistribution within the Ti–S framework, rather than a formal integer oxidation state. Because this descriptor is obtained from population analysis, it is used to discuss relative redistribution trends rather than to assign formal valence states. Accordingly, the sign and magnitude of the net charge indicate the direction and extent of electron accumulation or depletion relative to the reference state, rather than formal redox transitions. Under the same definition, the reverse deintercalation process would yield an opposite sign.

**Fig. 4:**
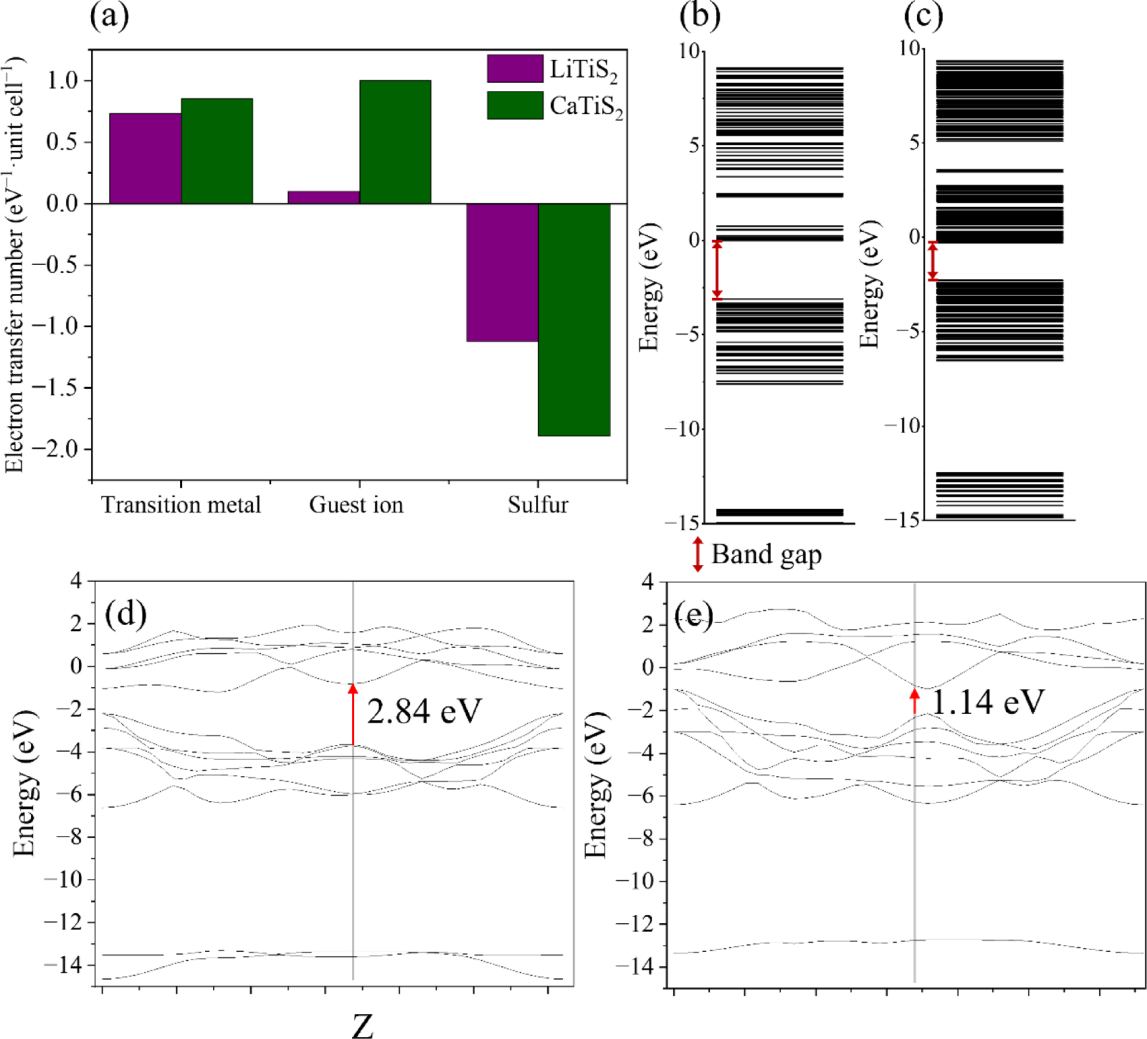
(**a**) Signed effective electron transfer (DV-Xα net charge) from guest ions, transition metals, and sulfur atoms in Li_13_Ti_26_S_48_ and Ca_13_Ti_26_S_48_; (**b**) MO diagram of LiTiS_2_; (**c**) MO diagram of CaTiS_2_; (**d**) band structure of bulk LiTiS_2_; and (**e**) band structure of bulk CaTiS_2_ (calculated using VASP and DV-Xα). In panel (**a**), positive and negative values indicate electron depletion and electron accumulation, respectively, within the adopted population-analysis definition.

The effective electron transfer from guest ions, transition metals, and sulfur atoms was quantified using the DV-Xα cluster method for both Li- and Ca-intercalated structures (Fig. 4). Li ions contribute only + 0.1 eV^− 1^·unit cell^− 1^, whereas Ca ions contribute + 1.0 eV^− 1^·unit cell^− 1^, indicating stronger charge donation from Ca^2+^ during interlayer ion diffusion. Ti atoms also show comparable-magnitude net-charge responses in CaTiS_2_ (+ 0.85 eV^− 1^·unit cell^− 1^) than in LiTiS_2_ (+ 0.73 eV^− 1^·unit cell^− 1^). However, the Ti-related values should be interpreted as a population-analysis descriptor of partial and delocalized redistribution, not as a direct readout of formal oxidation states, because Ti–S bonding involves substantial covalent electron sharing. Notably, the DV-Xα analysis was performed for Li_13_Ti_26_S_48_ and Ca_13_Ti_26_S_48_ (x ≈ 0.5) cluster models; therefore, even under an idealized ionic bookkeeping scheme, the average electron donation per Ti is fractional, and a one-to-one scaling of the Ti values with integer redox couples is not expected.

When sulfur atoms are explicitly considered, they exhibit significant negative charge accumulation (− 1.12 eV^− 1^·unit cell^− 1^ for LiTiS_2_ and − 1.89 eV^− 1^·unit cell^− 1^ for CaTiS_2_), demonstrating that sulfur acts as an important charge-compensation site within the Ti–S framework. In CaTiS_2_, the larger charge donation from Ca^2+^ is primarily reflected as increased electron accumulation on sulfur, rather than as a proportional change confined to Ti alone, whereas in LiTiS_2_, the smaller charge donation from Li^+^ results in a more modest overall redistribution. Accordingly, even if an idealized formal-redox bookkeeping assigns different Ti redox couples to Li and Ca intercalation, the DV-Xα net-charge values on Ti can remain of comparable magnitude because the additional charge is redistributed over the covalent Ti–S network (not localized exclusively on Ti).

The molecular-orbital (MO) diagrams obtained from DV-Xα cluster calculations reveal distinct electronic reorganization in LiTiS_2_ and CaTiS_2_. The diagrams clearly show bandgap narrowing from 2.84 to 1.14 eV upon Ca insertion. The red square regions in Fig. 4b and c mark the calculated MO gaps, highlighting the energy separation between the highest occupied and lowest unoccupied MOs. These electronic-structure changes reflect enhanced electronic delocalization induced by Ca^2+^ insertion during the charging process. Although the DV-Xα method is based on a finite cluster approximation, it reliably captures the relative evolution of electronic states associated with ion intercalation. Here, these values are interpreted as Kohn–Sham (model) gaps and are used as comparative descriptors of electronic-state evolution, rather than as quantitatively accurate quasiparticle band gaps. These electronic-structure changes reflect enhanced electronic delocalization induced by Ca^2+^ insertion during the charging process. Importantly, the bandgap values and their reduction trend are fully consistent with the bulk band-structure calculations performed using periodic DFT (VASP), as shown in Fig. 4d and e. This result indicates that Ca^2+^ intercalation promotes enhanced electronic delocalization and strengthens host–guest charge coupling, as reflected in the overall evolution of the electronic structure. Similarly, in Fig. 4d and e, the red arrows indicate the band-gap positions obtained from the bulk band-structure calculations using VASP, which are used to describe the relative separation between the valence and conduction bands. The bulk calculations (Fig. 4d and e) consistently reproduce the same qualitative trend, with CaTiS_2_ exhibiting a substantially narrower band gap than LiTiS_2_, thereby emphasizing the intercalation-induced band-gap narrowing rather than the absolute gap values (2.84 and 1.14 eV). The agreement between DV-Xα and periodic DFT calculations in capturing the relative band-gap evolution further demonstrates methodological consistency between localized and periodic electronic-structure analyses. These results are consistent with previous findings linking the band structure of TiS_2_ to its charge-accommodation capability^61–65^, confirming that Ca insertion enhances charge transfer and electronic conductivity.

### Overlap population between guest ions and TiS_2_ host material

**Fig. 5:**
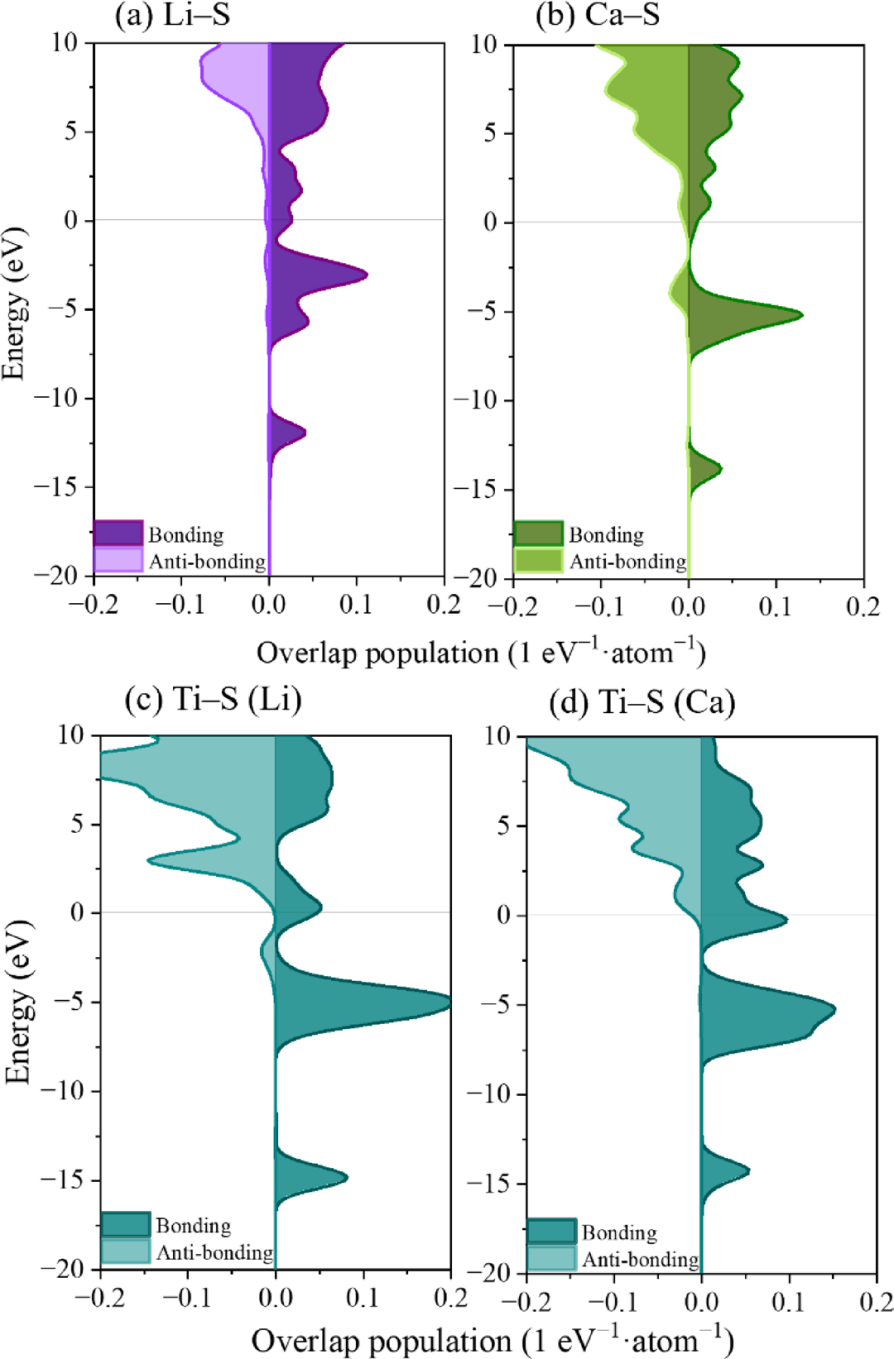
Overlap population diagrams for Li_13_Ti_26_S_48_ and Ca_13_Ti_26_S_48_: (**a**) Li–S bond; (**b**) Ca–S bond; (**c**) Ti–S bond in Li_13_Ti_26_S_48_; and (**d**) Ti–S bond in Ca_13_Ti_26_S_48_.

The charge-transfer analysis reveals that the behavior of electrons in the LiTiS_2_ and CaTiS_2_ structures are distinct. To clarify the ion diffusion behavior and host structural integrity, orbital-level bonding interactions were examined using the overlap population (OP) method, which quantifies the extent to which bonding electrons occupy the valence band and antibonding electrons occupy the conduction band^66–68^. Representative OP diagrams for the Li_13_Ti_26_S_48_ and Ca_13_Ti_26_S_48_ clusters are shown in Fig. 5, while results for other cluster sizes are provided in Figs. S5 and S6.

The bonding and antibonding electron densities for guest-ion–S and Ti–S interactions were quantified for both LiTiS_2_ and CaTiS_2_ clusters, as summarized in Table 1. For guest ion–S bonds, Li–S interactions exhibit a higher density of bonding electrons (0.183 eV^− 1^·atom^− 1^) and lower antibonding contribution (− 0.0074 eV^− 1^·atom^− 1^) than Ca–S interactions (0.175 and − 0.0193 eV^− 1^·atom^− 1^, respectively). This trend indicates that Ca–S bonds are weaker and more antibonding in nature, consistent with their higher ion mobility (Table S2). Conversely, for Ti–S bonds, CaTiS_2_ shows a slightly higher bonding electron density (0.346 vs. 0.338 eV^− 1^·atom^− 1^) and lower antibonding contribution (− 0.0042 vs. −0.0253 eV^− 1^·atom^− 1^), indicating reinforced Ti–S covalency upon Ca insertion.

**Table 1:** Bonding and antibonding electron densities in LiTiS_2_ and CaTiS_2_ clusters.

Structure	Bonding	Bonding electrons (eV^− 1^·atom^− 1^)	Anti-bonding electrons (eV^− 1^·atom^− 1^)
Li_13_Ti_26_S_48_	Li–S	0.183	−0.0074
	Ti–S	0.338	−0.0253
Ca_13_Ti_26_S_48_	Ca–S	0.175	−0.0193
	Ti–S	0.346	−0.0042

Taken together, these bonding metrics quantitatively confirm that Ca intercalation weakens direct guest–host (Ca–S) anchoring while simultaneously strengthening host-framework (Ti–S) interactions. Such dual behavior provides a mechanistic basis for the observed balance between the enhanced ion diffusion and structural stability of CaTiS_2_.

**Fig. 6:**
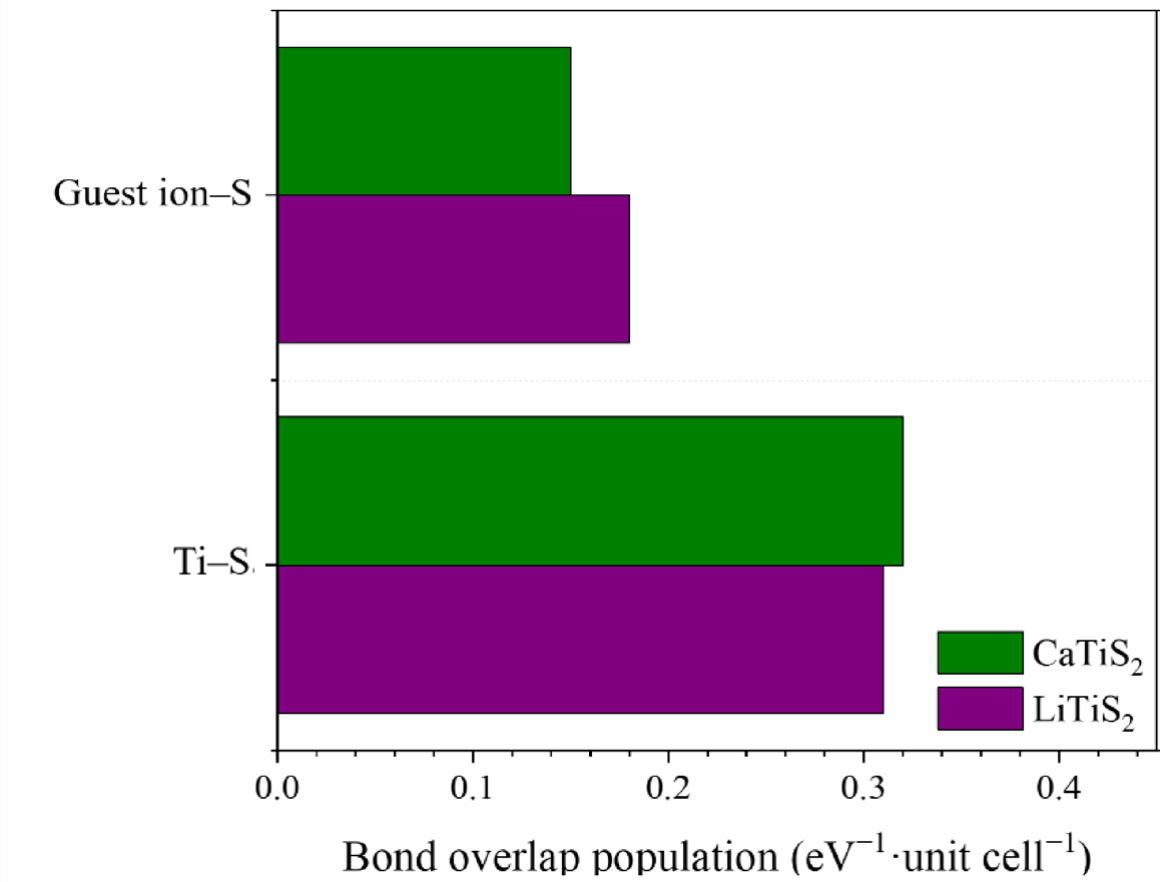
Effective covalent electron densities for guest ions, Ti, and S atoms in Li_13_Ti_26_S_48_ and Ca_13_Ti_26_S_48_.

To quantify the bond strength, Fig. 6 presents the integrated OP values. The effective covalent electron densities for Li–S and Ca–S bonds were 0.18 and 0.15 eV^− 1^·unit cell^− 1^, respectively, confirming weaker bonding of Ca with S atoms. For Ti–S bonds, the integrated OP values were 0.31 for LiTiS_2_ and 0.32 for CaTiS_2_, indicating a slight strengthening of Ti–S interactions in the Ca-intercalated structure. Previous research has reported an effective covalent electron count of 0.38 eV^− 1^·unit cell^− 1^ for Ti–S bonds in pristine TiS_2_^44^. Therefore, both LiTiS_2_ and CaTiS_2_ exhibited lower Ti–S covalency than pristine TiS_2_, reflecting the redistribution of charge induced by guest-ion insertion (Table S3).

### Adsorption and diffusion characteristics of LiTiS_2_ and CaTiS_2_

**Fig. 7:**
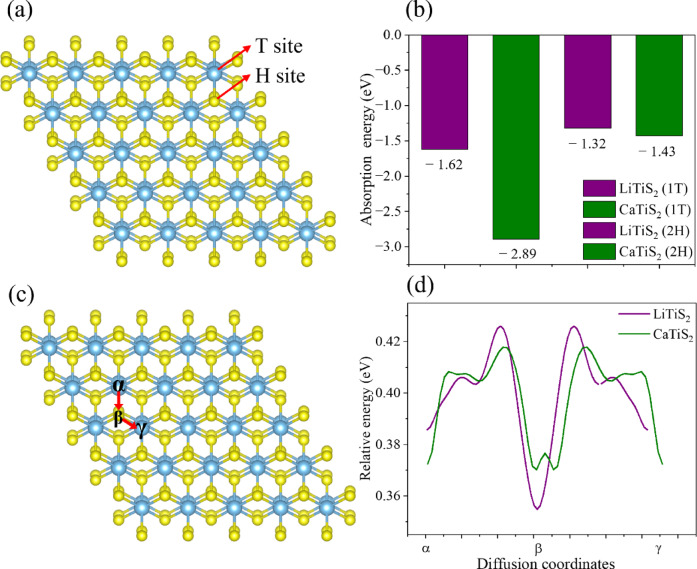
Adsorption and diffusion energy profiles of LiTiS_2_ and CaTiS_2_: (**a**) T and H sites within the TiS_2_ structure; (**b**) adsorption energies of LiTiS_2_ and CaTiS_2_ for 1T and 2H phases; (**c**) diffusion pathways (α, β, γ) in TiS_2_; and (**d**) relative diffusion energy profiles of LiTiS_2_ and CaTiS_2_ along α, β, and γ pathways.

Preferred ion-adsorption sites in the TiS_2_ lattice were identified at the T (top of triangle) and H (center of hexagon) positions, corresponding to local energy minima for guest-ion accommodation (Fig. 7a). Adsorption energies were evaluated for LiTiS_2_ and CaTiS_2_ in both the 1T and 2H polymorphs. LiTiS_2_ exhibits − 1.62 eV (T site) and − 1.32 eV (H site), while CaTiS_2_ shows more exothermic values of − 2.89 eV (T site) and − 1.43 eV (H site), indicating stronger binding for Ca and a clear preference for the T site in both systems (Fig. 7b)^69,70^. It should be noted that the adsorption energy discussed here represents a static thermodynamic quantity describing the stability of an ion at an equilibrium site, whereas the diffusion barrier reflects a dynamic kinetic process governed by the activation energy along a predefined migration pathway^71–73^. Therefore, a more negative adsorption energy does not necessarily imply a higher diffusion barrier, as the two quantities are governed by different aspects of the potential energy surface.

To examine ion mobility within the TiS_2_ lattice, three representative diffusion pathways were considered: α (T→H), β (T→T), and γ (H→H) (Fig. 7c)^74,75^. LiTiS_2_ exhibits a diffusion barrier of 0.07 eV along the α pathway, whereas CaTiS_2_ shows a substantially lower value of 0.03 eV, corresponding to a reduction of approximately 0.04 eV. Along the β and γ pathways, CaTiS_2_ also maintains lower barriers than LiTiS_2_, confirming that Ca ions migrate more easily within the layered TiS_2_ framework (Fig. 7d).

### Theoretical OCVs of LiTiS_2_ and CaTiS_2_

The bonding characteristics and lattice stability of LiTiS_2_ and CaTiS_2_ were used to evaluate their theoretical OCVs, which serve as a key descriptors of energy-storage efficiency and thermodynamic feasibility^76,77^. The OCV quantifies the maximum potential difference between electrodes under equilibrium and is thermodynamically linked to the Gibbs free energy change (ΔG) during ion intercalation (Table 2).

**Table 2:** Theoretical OCVs for LiTiS_2_ and CaTiS_2_.

Structure	Open circuit voltage (V)
LiTiS_2_	1.948
CaTiS_2_	1.383

The calculated theoretical OCVs indicate that LiTiS_2_ exhibits a higher value (1.948 V) than CaTiS_2_ (1.383 V) (Table 2). Here, the reported OCV represents an average intercalation voltage between the selected reference states used in the OCV expression, and it does not constitute a composition-resolved voltage plateau. A full voltage profile (plateau diagram) would require total energies of intermediate Ca_x_TiS_2_ configurations and competing Ca/vacancy orderings, which is beyond the scope of the present dataset. Rather than simply indicating weaker insertion tendency, this difference implies that LiTiS_2_ delivers a higher operating voltage and thus a higher energy density, while CaTiS_2_ may operate in a lower voltage regime that can help suppress electrolyte decomposition and side reactions during cycling.

The lower OCV of CaTiS_2_ is consistent with its more moderate electronic rearrangement and larger, yet structurally accommodated, lattice expansion upon ion insertion. These characteristics imply that CaTiS_2_ may experience reduced interfacial stress and potentially improved cycling stability, even though its energy output per charge is lower than that of LiTiS_2_. Additionally, because Ca^2+^ transfers two electrons per ion, CaTiS_2_ could achieve competitive charge storage despite operating in a lower voltage window.

Overall, these results indicate that LiTiS_2_ offers higher energy output, while CaTiS_2_ provides more favorable structural and interfacial behavior, reflecting a performance trade-off between energy density and long-term stability in Li^+^ and Ca^2+^ intercalation systems.

### Electrochemical performance of TiS_2_ based on capacity and ion transport

**Fig. 8:**
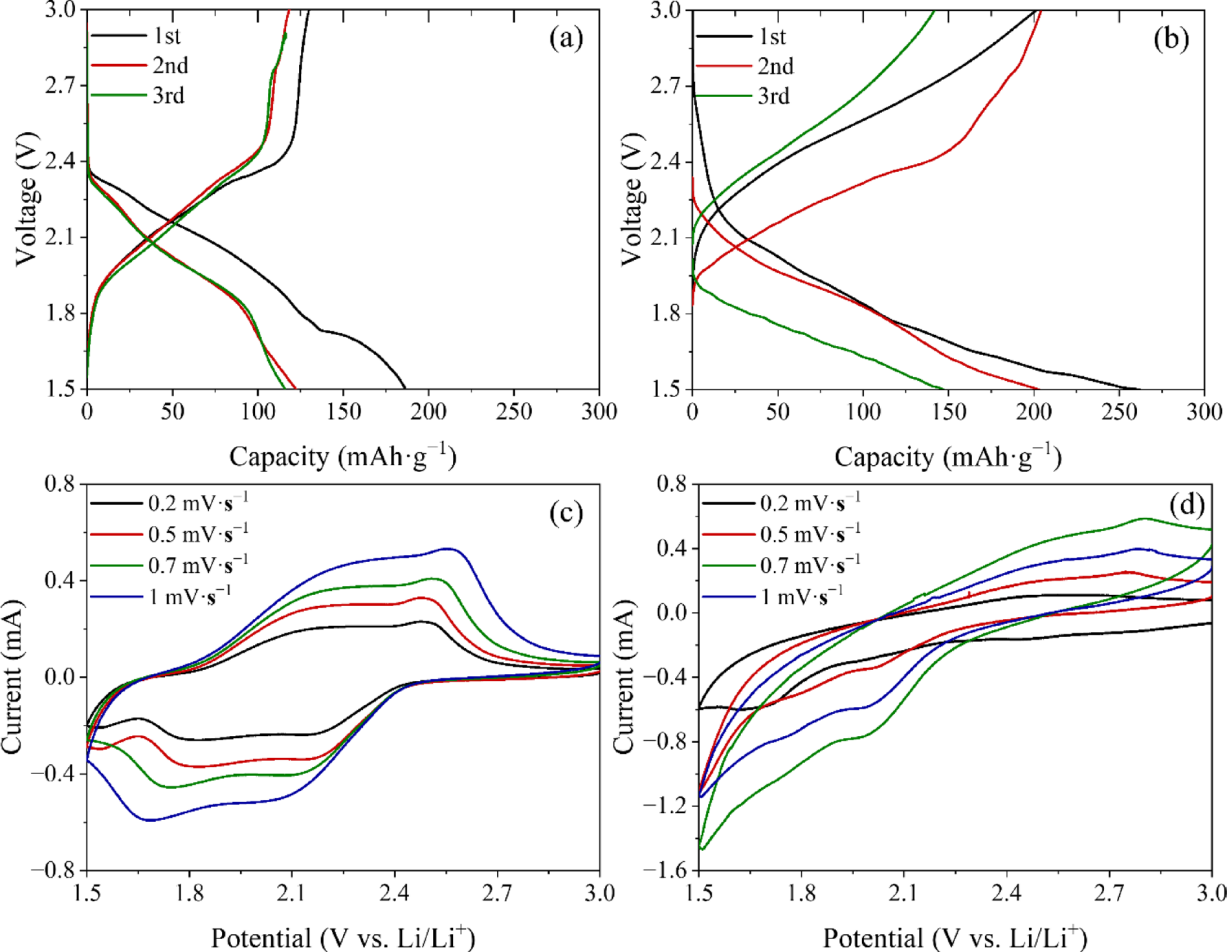
Electrochemical performance of TiS_2_ electrodes upon Li^+^ and Ca^2+^ intercalation. (**a**) Galvanostatic charge–discharge profiles of LiTiS_2_ for the first three cycles, showing stable voltage plateaus near 1.8 and 2.3–2.5 V. (**b**) Charge–discharge profiles of CaTiS_2_ for the first three cycles, exhibiting higher discharge capacity and distinct redox behavior with reduction ~ 2.0 V and oxidation near 2.8 V. (**c**) CV curves of LiTiS_2_ at scan rates of 0.2–1.0 mV·s^− 1^, confirming diffusion-controlled Li^+^ insertion/extraction. (**d**) CV curves of CaTiS_2_ at varying scan rates, where broader and shifted peaks reflect enhanced Ca^2+^ transport kinetics within the TiS_2_ host.

Galvanostatic charge–discharge profiles were analyzed to compare Li^+^ and Ca^2+^ intercalation behavior in TiS_2_. For Li intercalation, the first-cycle discharge capacity reached 134 mAh·g^− 1^ (Fig. 8a), while Ca intercalation achieved 201 mAh·g^− 1^, confirming the greater charge-storage capability of CaTiS_2_ (Fig. 8b). In addition, CaTiS_2_ exhibited a broader voltage plateau and reduced polarization compared to LiTiS_2_, suggesting distinct reaction mechanisms between the two systems. These curve differences imply that, even under identical conditions using lithium metal as both counter and reference electrodes, the observed electrochemical behavior arises from genuine Ca^2+^ intercalation rather than Li^+^ contamination.

This interpretation is consistent with previous in situ XRD studies, which directly confirmed reversible Ca^2+^ insertion into the TiS_2_ lattice without structural collapse^49^. In that report, characteristic diffraction peak shifts corresponding to (101) and (110) planes were observed during Ca^2+^ insertion and extraction, validating the intercalation mechanism. Taken together, the distinct voltage profiles obtained in this work and the structural evidence reported earlier strongly support the successful insertion of Ca^2+^ ions into the TiS_2_ host.

CV measurements also revealed distinct redox characteristics for Li and Ca insertion (Fig. 8c and d). In LiTiS_2_, a reduction peak corresponding to Li^+^ insertion appeared near 1.8 V, and oxidation peaks related to Li^+^ extraction were observed in the range of 2.3–2.5 V. In CaTiS_2_, the reduction peak was shifted to **~** 2.0 V, while the oxidation peak arose at **~** 2.8 V. These differences highlight the distinct redox behavior of Li^+^ and Ca^2+^ in the TiS_2_ lattice. CV measurements were conducted at scan rates of 0.2, 0.5, 0.7, and 1.0 mV·s^− 1^, and the corresponding increase in peak current with scan rate confirmed diffusion-controlled processes for both ions. The diffusion coefficients (D) of Li^+^ and Ca^2+^ were calculated using the Randles–Sevcik equation^78^:

$${i}_{p}=(2.69\times{10}^{5}){\mathrm{n}}^{3/2}\mathrm{A}{\mathrm{D}}^{1/2}\mathrm{C}{v}^{1/2}$$

where *i*_*p*_​ is the peak current (A), n is the number of electrons transferred, A is the electrode area (cm^2^), D is the diffusion coefficient (cm^2^·s^− 1^), C is the ion concentration (mol·cm^− 3^), and *v* is the scan rate (V·s^− 1^).

From the slopes of the *i*_*p*_​–*v*^1/2^ plots, the diffusion coefficient of Li^+^ was calculated to be 1.9 × 10^− 9^ cm^2^·s^− 1^, while that of Ca^2+^ was 3.1 × 10^− 9^ cm^2^·s^− 1^. The diffusion coefficients were determined by analyzing the anodic and cathodic peak currents at the respective redox potentials, confirming that Ca^2+^ exhibits faster ion transport than Li^+^ in the TiS_2_ host structure.

### Impact of Li^+^ and Ca^2+^ intercalation on rate capability and cycling stability

**Fig. 9:**
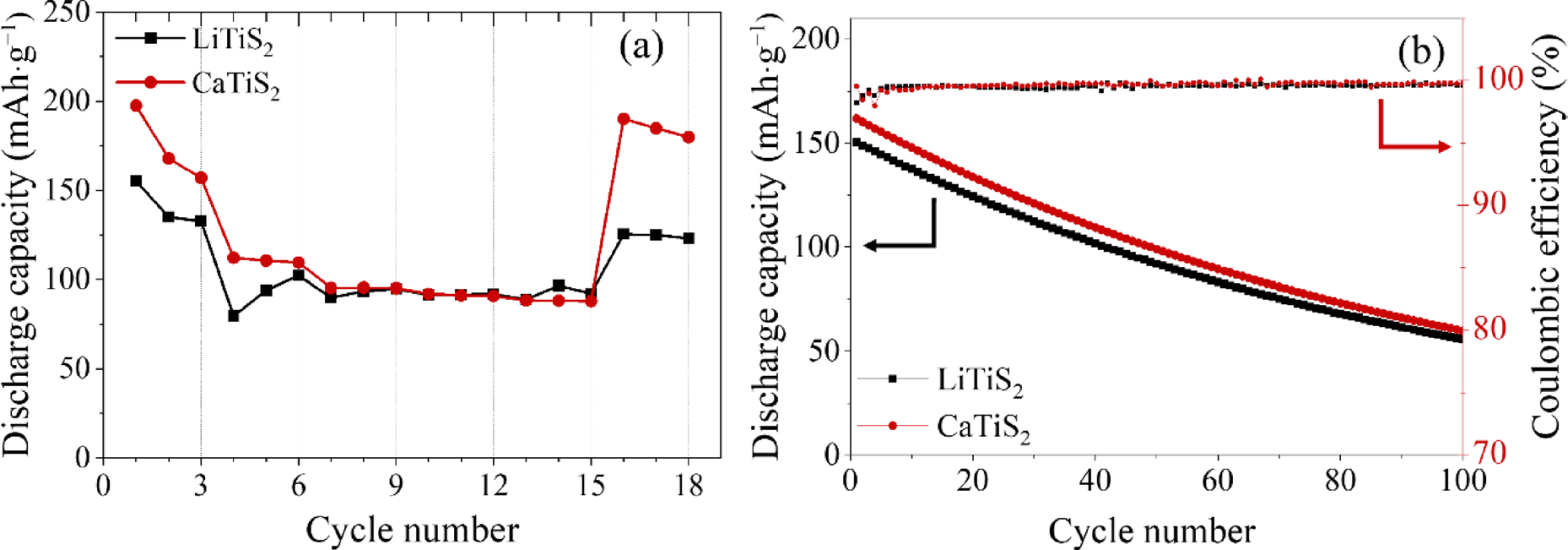
Cycling stability and rate capability of LiTiS_2_ and CaTiS_2_ electrodes. (**a**) Rate performance test, showing discharge capacities at varying current densities and subsequent recovery when the current returns to the initial condition. CaTiS_2_ exhibits better capacity retention than LiTiS_2_. (**b**) Long-term cycling performance over 100 cycles, where both electrodes show gradual capacity fading but maintain stable coulombic efficiency (~ 99% ± 1%). CaTiS_2_ delivers slightly higher discharge capacities and comparable stability under identical conditions, confirming favorable structural tolerance to repeated intercalation.

The rate capability and long-term cycling performance of LiTiS_2_ and CaTiS_2_ electrodes were systematically evaluated. In the rate capability test (Fig. 9a), both electrodes exhibited reversible capacity recovery after returning to the initial current density. The capacity retention ratio after rate testing was 88.5% for LiTiS_2_ and 96.3% for CaTiS_2_, indicating that Ca intercalation leads to a higher rate capability than Li intercalation.

The cycling performance over 100 cycles is shown in Fig. 9b. After prolonged cycling, the capacity retention was 35.4% for LiTiS_2_ and 36.6% for CaTiS_2_, demonstrating that both systems possess comparable long-term stability under identical conditions. Despite the similar overall retention, CaTiS_2_ consistently delivered slightly higher discharge capacities than LiTiS_2_ during the cycling test. Coulombic efficiency remained stable at ~ 99% ± 1% throughout the cycles for both electrodes, confirming good reversibility of the intercalation process.

The gradual decrease in capacity observed during cycling can be attributed to structural degradation of the TiS_2_ host, partial loss of the active material, and accumulation of resistive interfacial layers over repeated insertion/extraction. Nevertheless, the results confirm that Ca intercalation leads to a higher rate capability and cycling stability comparable to that of TiS_2_ intercalated with Li.

## Discussion

The intercalation of multivalent cations into layered hosts has long been regarded as challenging owing to sluggish diffusion and structural instability. In particular, Ca^2+^ has often been considered unsuitable owing to its larger ionic radius and strong coulombic interactions. This study challenges these assumptions, demonstrating through a combined theoretical–experimental approach that TiS_2_ can host Ca^2+^ ions both coherently and effectively while maintaining its layered integrity.

Theoretical analyses revealed three key features underlying this behavior. First, Ca^2+^ insertion resulted in a noticeable interlayer expansion that was greater than that of Li^+^, which widened the diffusion channels but preserved crystallographic coherence through reinforced Ti–S bonding. This delicate balance between weaker guest anchoring and stronger host covalency created a previously unexplored insertion regime for divalent systems. Second, electronic structure calculations showed that Ca insertion markedly increased the DOS near the Fermi level and narrowed the bandgap, reflecting enhanced Ti orbital hybridization and suggesting improved electronic conductivity and redox activity. Third, orbital overlap analysis revealed that while Ca–host bonding relatively weakened, Ti–S covalency strengthened, enabling facile ion mobility without structural breakdown. From a mechanistic perspective, the coexistence of strong adsorption and fast diffusion for Ca^2+^ originates from a decoupling between static ion stabilization and dynamic migration processes, as reported in previous theoretical studies^71–73^. While adsorption energy reflects the thermodynamic depth of local binding sites dominated by electrostatic interactions, ion diffusion is governed by the saddle-point energy along the migration pathway, which is strongly influenced by host covalency and electronic screening.

The experimental evidence supported these theoretical predictions. CV testing confirmed higher redox potentials for Ca insertion, while the kinetic analyses revealed unexpectedly fast ion transport consistent with the calculated low diffusion barriers. Long-term cycling stability measurements also demonstrated gradual rather than abrupt capacity decay, implying that the Ti–S framework remains mechanically resilient during repeated intercalation. Together, these results establish a new paradigm in divalent-ion intercalation: Ca^2+^ simultaneously weakens guest binding and strengthens host bonding, modifies band topology while expanding interlayers, and enhances charge transfer while minimizing diffusion barriers.

Beyond TiS_2_, this work contributes a broader design principle for multivalent-ion hosts: maintaining weak guest–host binding to promote diffusion, ensuring strong host frameworks to resist degradation, enhancing DOS for improved conductivity, and controlling interlayer spacing to balance mobility and stability. The combined use of DV-Xα and DFT methodologies proved particularly powerful for correlating orbital-level interactions with experimentally measurable electrochemical behavior, underscoring the value of theory as both predictive and explanatory. These findings establish Ca-intercalated TiS_2_ as a robust prototype for advancing next-generation CIBs.

In the broader context of layered chalcogenide electrodes, the behavior observed in TiS_2_ can be discussed alongside previous lithium-ion studies on MoS_2_ and related materials. Prior work on MoS_2_ has shown that Li^+^ intercalation is often accompanied by pronounced interlayer expansion, electronic-structure modulation, and, in some cases, phase-transition-related structural rearrangements^79,80^. In comparison, the present results suggest that TiS_2_ exhibits a more coherently balanced response during Ca^2+^ insertion, where interlayer expansion, electronic rehybridization, and host-framework stabilization occur without severe structural disruption. From this perspective, the present results point to the need and potential for extending integrated computational–electrochemical studies to a wider range of layered materials, enabling a more systematic correlation between electronic structure, bonding characteristics, and experimentally observed intercalation behavior in multivalent-ion systems.

## Conclusions

This study demonstrates that Ca^2+^ intercalation into TiS_2_ establishes a unique interplay between weakened guest anchoring, reinforced host covalency, and enhanced electronic activity. The cooperative effect of these factors enables rapid ion diffusion, efficient charge transfer, and high structural resilience, all of which overcome the traditional limitations of divalent-ion systems. By combining theoretical modeling and electrochemical validation, the work provides atomistic insight into how Ca^2+^ modifies the Ti–S lattice and electronic framework, thereby redefining the criteria for viable multivalent-ion hosts.

Future research should focus on extending this strategy to other layered chalcogenides and exploring electrolyte compositions that further stabilize Ca^2+^ transport. Particular attention should be given to interfacial phenomena, where desolvation kinetics and charge-transfer barriers often dictate overall performance. Additionally, the integration of in situ characterization techniques with first-principles modeling will be essential to capture real-time structural dynamics during intercalation. By advancing this multiscale understanding, the design of durable, high-performance CIBs—and more broadly, next-generation multivalent systems—can be achieved with improved predictability and efficiency.

A quantitatively resolved thermodynamic assessment of Ca_x_TiS_2_ requires convex hull analysis across a broad Ca concentration range to evaluate phase stability/metastability and to test whether the electrochemical response is governed by solid-solution behavior or phase separation. Likewise, constructing a composition-resolved voltage profile and correlating voltage features with deintercalation-induced structural rearrangements would be necessary to quantitatively link structural evolution to cycling stability. Because these composition-dependent analyses require extensive additional sampling beyond the present dataset, they are identified here as key directions for future work.

## Supplementary Information

Below is the link to the electronic supplementary material.

Supplementary Material 1

## Data Availability

The datasets generated and analyzed during the current study are available from the corresponding author on reasonable request.
