# Supplementary material for: Atomistic and electronic insights into Ca2+ and Li+ intercalation in TiS2: a first-principles approach supported by electrochemical validation
Source: Sci Rep. 2026 Mar 23;16:14605. doi: 10.1038/s41598-026-42087-w (PMC13153370; doi:10.1038/s41598-026-42087-w)
Supplement: Supplementary file 1 — Supplementary Material 1 [file 41598_2026_42087_MOESM1_ESM.docx]

Supporting Information

**Atomistic and Electronic Insights into Ca^2+^ and Li^+^ Intercalation in TiS_2_: A First-Principles Approach Supported by Electrochemical Validation**

Seunga Yang^1^, SangYup Lee^1^, Paul Maldonado Nogales^1^, Yangsoo Kim^2^*, and Soon-Ki Jeong^1^*

^1^Department of Energy Engineering, Soonchunhyang University, Soonchunhyang-ro 22-gil, Sinchang-myeon, Asan-si 31538, Chungcheongnam-do, Republic of Korea

^2^Korea Basic Science Institute, Jeonju Center, Jeonju-si, Jeollabuk-do 54907, Republic of Korea

*Corresponding authors: kimyangsoo@kbsi.re.kr (Y.K.) and hamin611@sch.ac.kr (S.-K.J.)


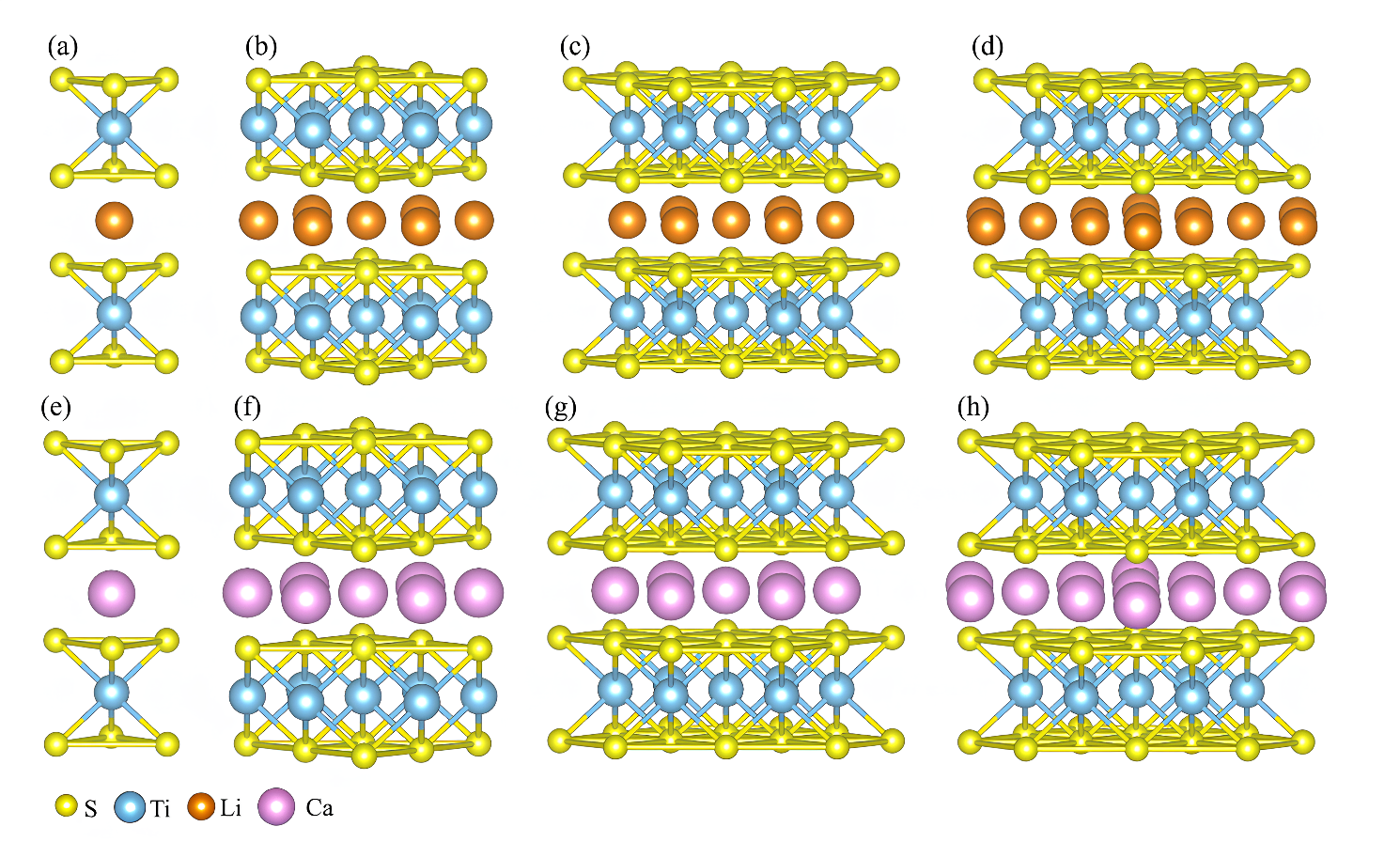


**Figure S1.** Structural schematics of (a), (e) XTi_2_S_12_; (b), (f) X_7_Ti_14_S_24_; (c), (g) X_7_Ti_14_S_48_ (d); and (h) X_13_Ti_14_S_48_ with guest ions situated between two layers in the (100) plane (X represents Li or Ca).


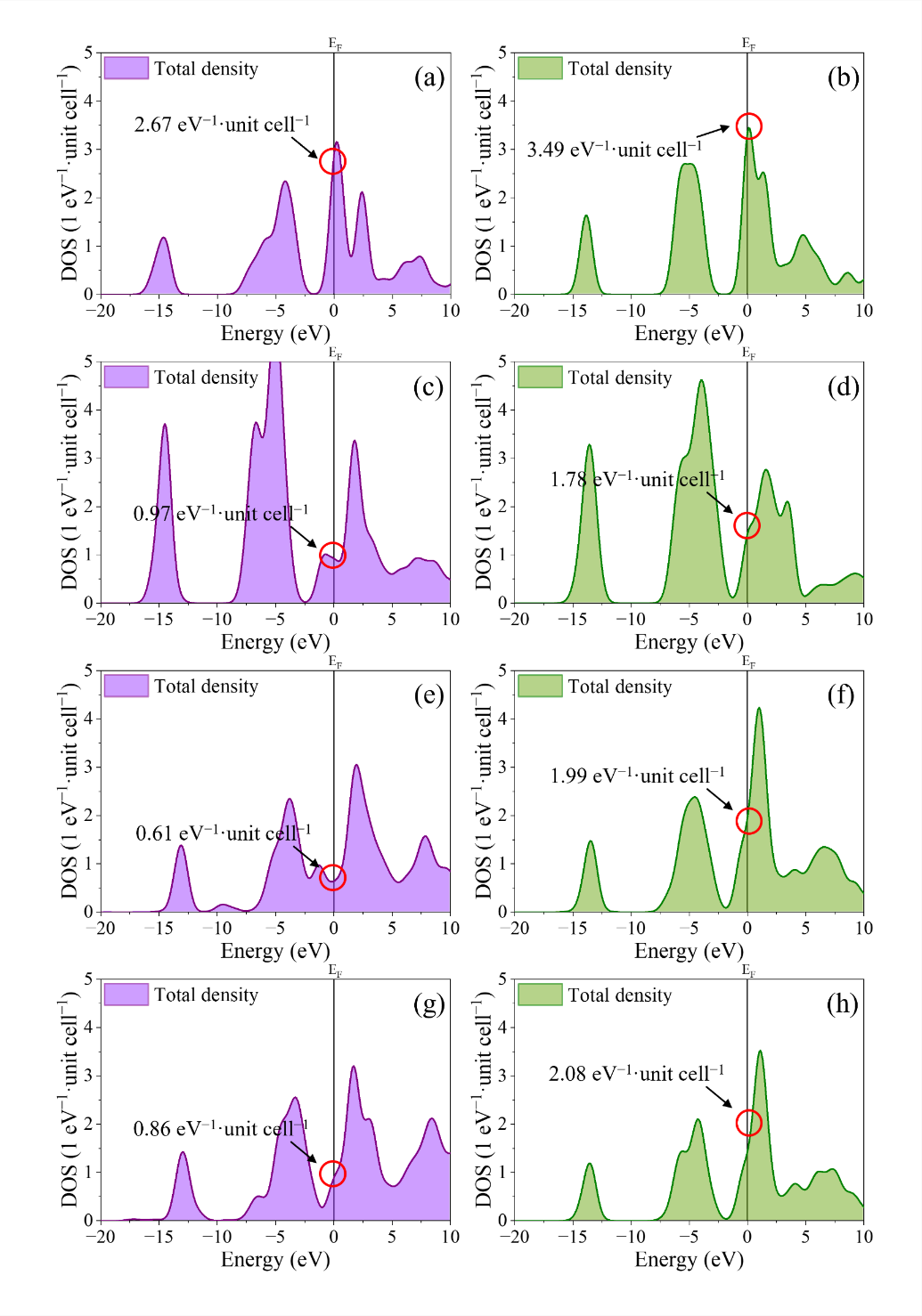


**Figure S2.** Comparison of the DOS: total DOS for the Li-intercalated structure (a) LiTi_2_S_12_, (c) Li_7_Ti_14_S_24_, (e) Li_7_Ti_14_S_48_, and (g) Li_13_Ti_14_S_48_, and the Ca-intercalated (b) CaTi_2_S_12_, (d) Ca_7_Ti_14_S_24_, (f) Ca_7_Ti_14_S_48_, and (h) Ca_13_Ti_14_S_48_.


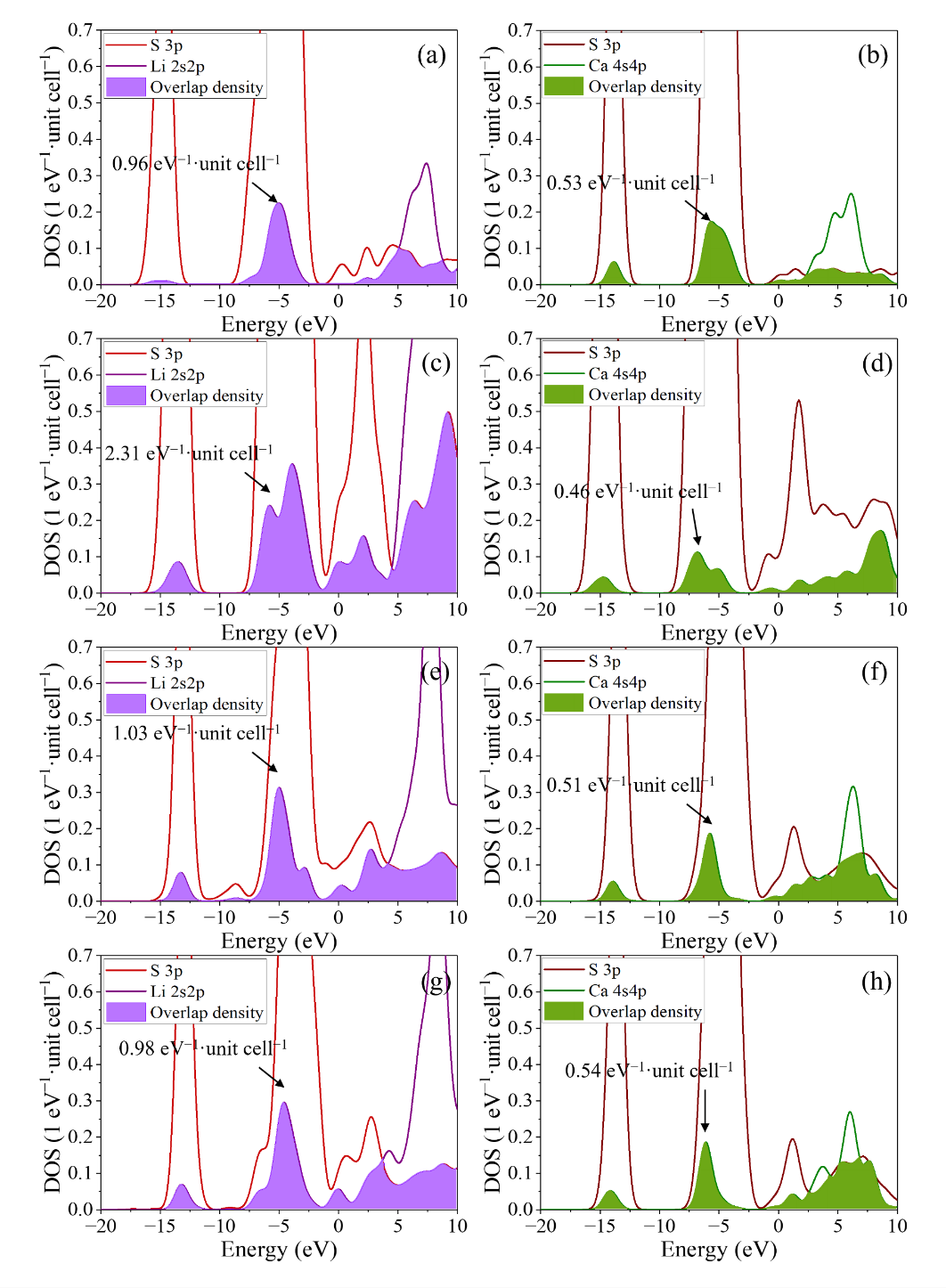


**Figure S3.** Comparison of partial DOS between partial DOS of the guest-ion sp and S 3p orbitals in (a) LiTi_2_S_12_, (c) Li_7_Ti_14_S_24_, (e) Li_7_Ti_14_S_48_, and (g) Li_13_Ti_14_S_48_, and the Ca-intercalated (b) CaTi_2_S_12_, (d) Ca_7_Ti_14_S_24_, (f) Ca_7_Ti_14_S_48_, and (h) Ca_13_Ti_14_S_48_.


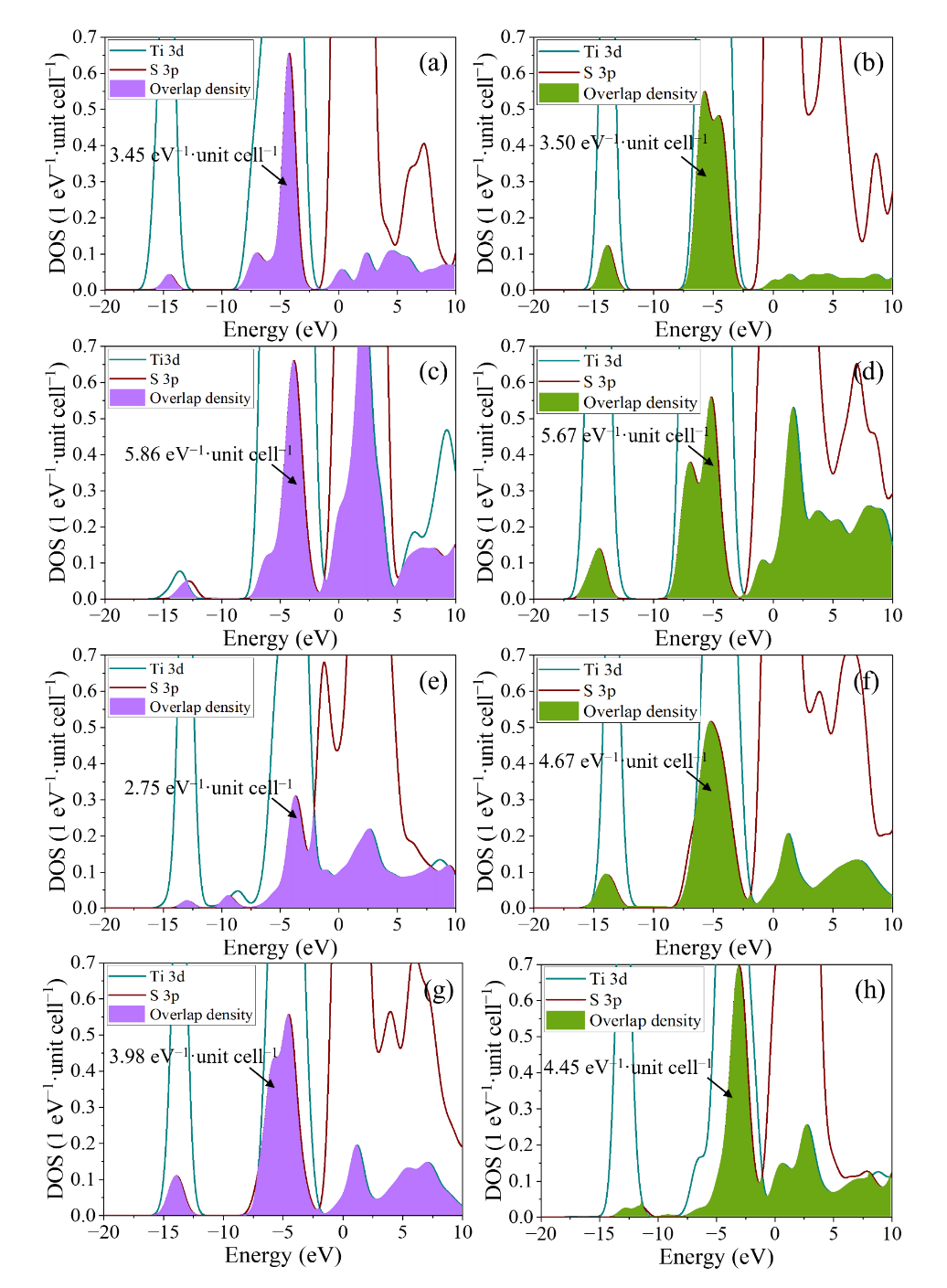


**Figure S4.** Comparison of partial DOS between the S 3p and Ti 3d orbitals in (a) LiTi_2_S_12_, (c) Li_7_Ti_14_S_24_, (e) Li_7_Ti_14_S_48_, and (g) Li_13_Ti_14_S_48_, and the Ca-intercalated (b) CaTi_2_S_12_, (d) Ca_7_Ti_14_S_24_, (f) Ca_7_Ti_14_S_48_, and (h) Ca_13_Ti_14_S_48_.

**
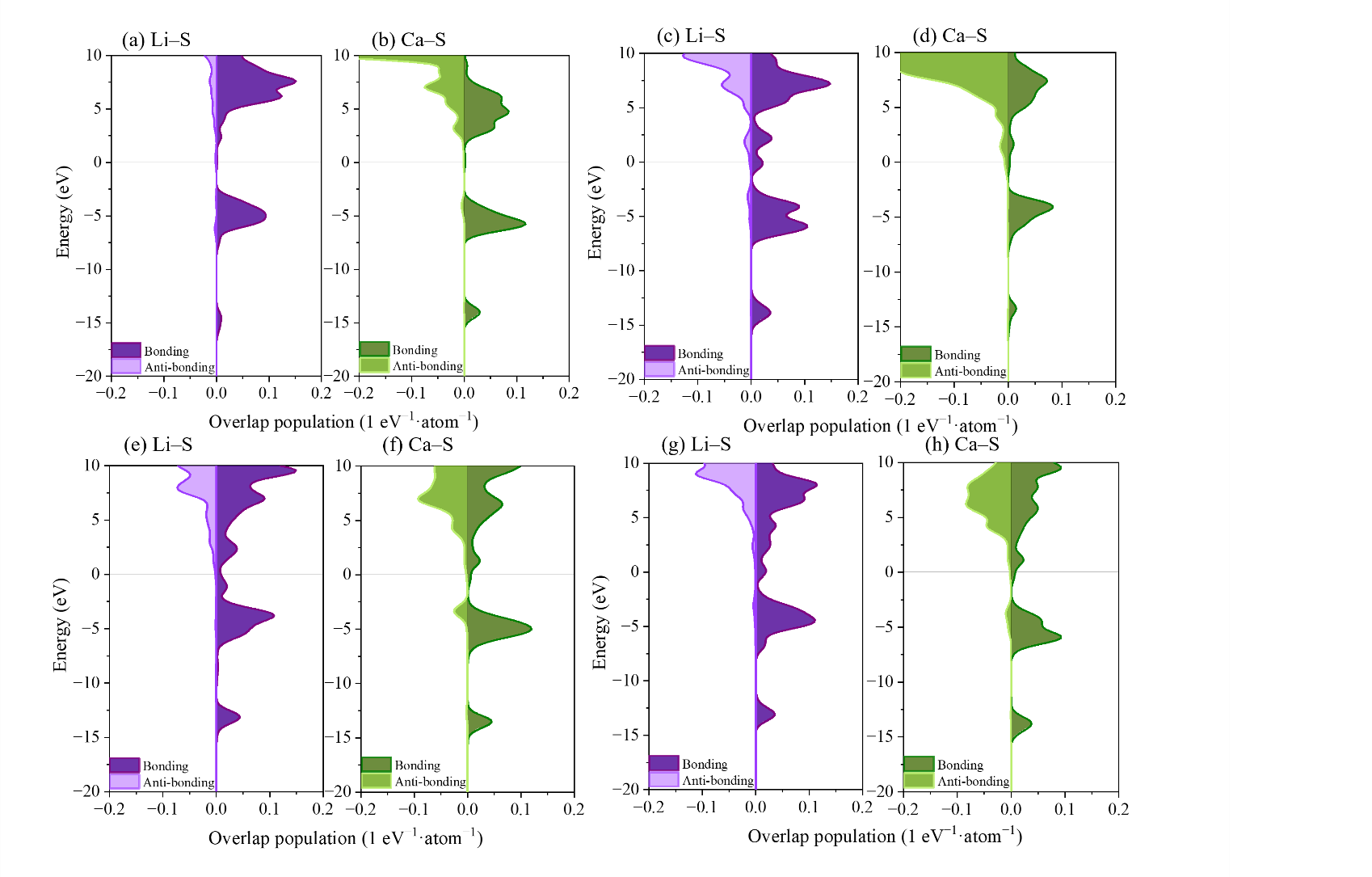
**

**Figure S5.** Overlap population diagrams for Li–S and Ca–S bonds in (a), (b) XTi_2_S_12_; (c), (d) X_7_Ti_14_S_24_; (e), (f) X_7_Ti_14_S_48_; and (g), (h) X_13_Ti_14_S_48_.

**
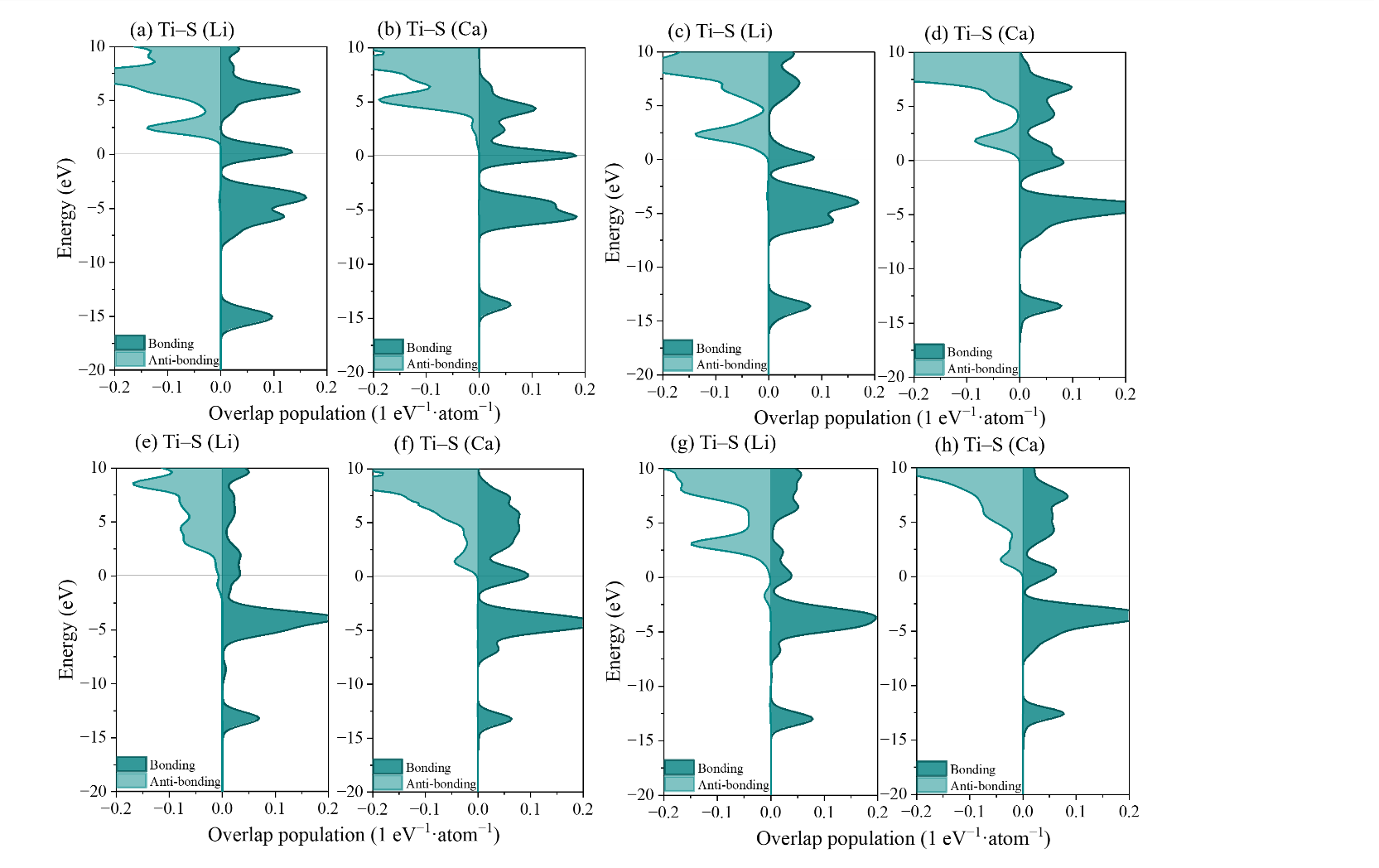
**

**Figure S6.** Overlap population diagrams for Ti–S bond in (a), (b) XTi_2_S_12_; (c), (d) X_7_Ti_14_S_24_; (e), (f) X_7_Ti_14_S_48_; and (g), (h) X_13_Ti_14_S_48_.

**Table S1.** Electron transfer number in LiTiS_2_ and CaTiS_2_ clusters.

| **Structure** | **Atom** | **Electron transfer number (eV^−1^**·**unit cell^−1^)** |
| --- | --- | --- |
| **LiTi_2_S_12_** | Li | 0.41 |
|  | Ti | 1.14 |
|  | S | −1.57 |
| **CaTi_2_S_12_** | Ca | 1.47 |
|  | Ti | 1.26 |
|  | S | −1.73 |
| **Li_7_Ti_14_S_24_** | Li | 0.12 |
|  | Ti | 0.73 |
|  | S | −0.46 |
| **Ca_7_Ti_14_S_24_** | Ca | 1.18 |
|  | Ti | 1.01 |
|  | S | −1.09 |
| **Li_7_Ti_14_S_48_** | Li | 0.08 |
|  | Ti | 0.91 |
|  | S | −0.32 |
| **Ca_7_Ti_14_S_48_** | Ca | 1.21 |
|  | Ti | 1.09 |
|  | S | −0.98 |
| **Li_13_Ti_14_S_48_** | Li | 0.23 |
|  | Ti | 0.76 |
|  | S | −0.34 |
| **Ca_13_Ti_14_S_48_** | Ca | 1.27 |
|  | Ti | 1.05 |
|  | S | −1.03 |

**Table S2.** Bonding and antibonding electron densities in LiTiS_2_ and CaTiS_2_ clusters.

| **Structure** | **Bonding** | **Bonding electrons**  **(eV^−1^**·**atom^−1^)** |  | **Anti-bonding electrons**  **(eV^−1^**·**atom^−1^)** |
| --- | --- | --- | --- | --- |
| **LiTi_2_S_12_** | Li–S | 0.158 |  | −0.0055 |
|  | Ti–S | 0.332 |  | −0.0022 |
| **CaTi_2_S_12_** | Ca–S | 0.131 |  | −0.0039 |
|  | Ti–S | 0.381 |  | −0.0026 |
| **Li_7_Ti_14_S_24_** | Li–S | 0.199 |  | −0.0095 |
|  | Ti–S | 0.369 |  | −0.0037 |
| **Ca_7_Ti_14_S_24_** | Ca–S | 0.161 |  | −0.0067 |
|  | Ti–S | 0.388 |  | −0.0004 |
| **Li_7_Ti_14_S_48_** | Li–S | 0.182 |  | −0.0027 |
|  | Ti–S | 0.316 |  | −0.0078 |
| **Ca_7_Ti_14_S_48_** | Ca–S | 0.183 |  | −0.0221 |
|  | Ti–S | 0.339 |  | −0.0009 |
| **Li_13_Ti_14_S_48_** | Li–S | 0.179 |  | −0.0052 |
|  | Ti–S | 0.335 |  | −0.0106 |
| **Ca_13_Ti_14_S_48_** | Ca–S | 0.165 |  | −0.0134 |
|  | Ti–S | 0.351 |  | −0.0007 |

**Table S3.** Bond overlap populations in LiTiS_2_ and CaTiS_2_ clusters.

| **Structure** | **Bonding** | **Bond overlap population number (eV^−1^**·**unit cell^−1^)** |
| --- | --- | --- |
| **LiTi_2_S_12_** | Li–S | 0.13 |
|  | Ti–S | 0.34 |
| **CaTi_2_S_12_** | Ca–S | 0.07 |
|  | Ti–S | 0.33 |
| **Li_7_Ti_14_S_24_** | Li–S | 0.14 |
|  | Ti–S | 0.32 |
| **Ca_7_Ti_14_S_24_** | Ca–S | 0.07 |
|  | Ti–S | 0.35 |
| **Li_7_Ti_14_S_48_** | Li–S | 0.18 |
|  | Ti–S | 0.34 |
| **Ca_7_Ti_14_S_48_** | Ca–S | 0.11 |
|  | Ti–S | 0.33 |
| **Li_13_Ti_14_S_48_** | Li–S | 0.19 |
|  | Ti–S | 0.34 |
| **Ca_13_Ti_14_S_48_** | Ca–S | 0.11 |
|  | Ti–S | 0.38 |
